# Integration of microRNA and mRNA analyses depicts the potential roles of *Momordica charantia* saponin administration in insulin resistance of juvenile common carp (*Cyprinus carpio*) fed with a high-starch diet

**DOI:** 10.3389/fmolb.2023.1054949

**Published:** 2023-04-05

**Authors:** Ze Fan, Liansheng Wang, Chenhui Li, Di Wu, Jinnan Li, Haitao Zhang, Siwei Xiong, Linghong Miao, Xianping Ge, Zhengwei Li

**Affiliations:** ^1^ Key Laboratory of Aquatic Animal Diseases and Immune Technology of Heilongjiang Province, Heilongjiang River Fisheries Research Institute, Chinese Academy of Fishery Sciences, Harbin, China; ^2^ Key Laboratory of Aquatic, Livestock and Poultry Feed Science and Technology in South China, Ministry of Agriculture and Rural Affairs, Guangdong Evergreen Feed Industry Co., Ltd., Zhanjiang, China; ^3^ Tianjin Agricultural University, Tianjin, China; ^4^ Key Laboratory of Freshwater Fisheries and Germplasm Resources Utilization, Ministry of Agriculture and Rural Affairs, Freshwater Fisheries Research Center, Chinese Academy of Fishery Sciences, Wuxi, China; ^5^ Heilongjiang Aquatic Animal Resource Conservation Center, Heilongjiang, China

**Keywords:** *Cyprinus carpio*, insulin resistance, *Momordica charantia* saponins, microRNA, target-regulating

## Abstract

**Background:** The regulation of target gene mRNA mediated by microRNA may play an important role in glucose metabolism in fish. Previous research findings of our research group revealed that *Momordica charantia* saponin (MS) administration in a high-starch diet could improve insulin resistance of common carp through renovating insulin signaling pathways, whose fundamental mechanisms have remained unknown by far. To reveal this potential mechanism, we aimed to investigate the difference in miRNA and mRNA expression profiles between common carp fed with high-starch diets containing MS (HS_MS1 and HS_MS2) and common carp fed with high-starch (HS) diets.

**Results:** Through miRNA deep-sequencing, 10 significantly differentially expressed miRNAs in HC and HS_MS1, including one upregulated and nine downregulated miRNAs, were identified, whereas 10 significantly differentially expressed miRNAs in HC and HS_MS2, including four upregulated and six downregulated miRNAs, were identified. These miRNAs may not only be involved in the regulation of insulin signaling pathways and insulin resistance in common carp but also be the markers for liver insulin resistance in MS therapy for the remission of insulin resistance. This study identified 10 potential known miRNAs, namely, ccr-miR-10b, ccr-miR-122, ccr-miR-143, ccr-miR-146a, ccr-miR-155, ccr-miR-16c, ccr-miR-200a, ccr-miR-29a, ccr-miR-34, and ccr-miR-375, as candidates participating in modulating the liver insulin resistance. According to the biopathway enrichment analysis of the 252 target genes using the KEGG classical biopathway database, the relative expression levels of gsk3bb, pik3r1, and pik3r3b were analyzed using RNA-seq. Compared to the HC group, a significant decrease in the relative expression levels of pik3r1 and pik3r3b was observed in HS_MS1 and HS_MS2 groups (*p* < 0.05). This study raised a presumption of the presence of ccr-miR-29a targeting pik3r1 or ccr-miR-143 targeting pik3r3 playing likely roles in *Momordica charantia* saponins remitting the liver insulin resistance.

**Conclusion:** The findings will further deepen the understanding of the carbohydrate metabolism of common carp and provide an important scientific basis for the application of *Momordica* saponins as functional nutrients to alleviate insulin resistance of fish in fish culture.

## Introduction

In comparison to carnivorous fishes, omnivorous common carp could facilitate slightly better use of carbohydrate in feed, but the “congenital diabetic constitution” of “hyperglycemia intolerance” still remains the key factor hindering their thorough use of carbohydrate (Polakof et al., 2012; Kamalam et al., 2017). Our previous study reported that the blood glucose content peaked at 3 h after high-dose glucose was injected to common carp, while the insulin content peaked at 6 h after injection, lagging behind the blood glucose response, and concurrently, the inhibition of glucagon secretion by the increase in blood glucose and insulin levels was weakened (Cheng et al., 2018). Based on these findings, the hypothesis that there exists insulin resistance in the common carp liver was established. Therefore, in production practice, it is necessary to explore the underlying mechanism of insulin resistance in fish and the functional nutrients or aquaculture strategies that can alleviate insulin resistance in fish.

In many cellular processes, microRNAs (miRNAs) are deemed key regulators of post-transcriptional gene silencing. Studies on the composition and function of microRNA in common carp have begun to accumulate. Ma et al. (2021) reported that miR-153b-3p could regulate spermatogenesis by targeting amh in common carp. Yan et al. (2012) found that miR-1, miR-21, miR-26a, miR-27a, miR-133a-3p, miR-206, miR-214, and miR-222 had obviously differential expression changes, and it was speculated that these miRs might play a crucial role in the overall development process and muscle development process of common carp. Chen et al. (2020) found that 23 differentially expressed microRNAs targeted NF-κB signaling pathway, Janus kinase-signal transducers and activators of transcription (JAK-STAT) signaling pathway, mitogen-activated protein kinase (MAPK) signaling pathway, Th1 and Th2 cell differentiation, and toll-like receptor signaling pathway to mediate spleen inflammatory injury in common carp under cadmium stress. When common carp suffered from cadmium stress, miRNAs mediated inflammation-immunosuppressive injury caused by Cd in the spleens by targeted regulation of nuclear factor kappa-light-chain-enhancer of activated B cell (NF-κB) signaling pathway, JAK-STAT signaling pathway, MAPK signaling pathway, Th1 and Th2 cell differentiation, and toll-like receptor signaling pathway (Chen et al., 2020). Remarkably, the functional identification of 134 miRs for common carp included in the miRBase database was mainly limited to growth and development and immunocompetence. The impaired insulin signaling pathway is an important factor in the occurrence of insulin resistance. The most important link in the insulin signal transduction pathway is through the phosphoinositide-3-kinas/proteinkinase B (PI3K/AKT) signaling pathway to regulate blood glucose (Sun et al., 2021). To date, previous studies on insulin signaling pathways in fish mainly focused on protein-coding genes such as signaling molecules. Nevertheless, there is little information available on the role of non-coding sequences such as miRs which are closely related to gene expression abnormalities. Therefore, for the purpose of alleviating the insulin resistance of common carp in basic research, it is necessary to explore the role of the targeted regulatory relationship between miRs and the insulin signal transduction pathway with regard to carbohydrate metabolism in fish.

With the deepening of research, the studies on fish carbohydrate nutrition gradually turns to the nutrient regulatory level. Plant extracts such as saponins and flavonoids have the function of assisting to improve the absorption capacity of glucose metabolism (Zhao et al., 2020; Ning et al., 2022), but there is no relevant report on the study of fish carbohydrate nutrition. The study on the hypoglycemic activity of *Momordica charantia* saponins has always been a hot spot in the research on natural active ingredients (Zheng et al., 2021). A measure of 300 μg/mL *Momordica charantia* saponins can not only obviously improve the activity of insulin-resistant cells but also increase the consumption of glucose, which contributes to alleviating the symptoms of insulin resistance (You et al., 2014). Likewise, our previous study also demonstrated that 1600 mg/kg of *Momordica charantia* saponins for juvenile common carp could attenuate the liver insulin resistance by enhancing glycolysis ability, promoting the glycogen synthesis and inhibiting the gluconeogenesis through activating the PI3K/AKT signaling pathway (Fan et al., 2022). Thereat, the fundamental mechanism of *Momordica charantia* saponins regulating the insulin signal transduction pathway need to be dug deep in fish.

On the basis of the previous study, the expression profiles of differentially expressed miRNAs and mRNAs between common carp fed with high-starch diets containing *Momordica charantia* and common carp fed with high-starch diets were reaped using the RNA sequencing strategy, and the miRNAs related to insulin resistance were identified. Furthermore, the miRNA target gene prediction analysis, function analysis, and miRNA–mRNA integration analysis gave assistance to predicting signaling pathways related to insulin resistance. The findings will broaden our horizon of the intricate and underlying mechanism on “congenital diabetic constitution” in fish and contribute to further developing the high-carbohydrate and low-protein diets for common carp.

## Materials and methods

### Ethics statement

All animal procedures in this study were conducted according to the guidelines for the care and use of laboratory animals of Heilongjiang River Fisheries Research Institute (CAFS). The animal studies were reviewed and approved by the Committee for the Welfare and Ethics of Laboratory Animals of Heilongjiang River Fisheries Research Institute.

### Experimental procedures and sample collection

In consideration of NRC (2011) and our previous findings (Fan et al., 2022), 1.6 g/kg (HS_MS1) and 6.4 g/kg (HS_MS2) administration levels in high-starch diets were chosen for further study. *Momordica charantia* saponin-free diets with 300 g/kg starch levels were designated as the control diet (HS) containing 304.8 g/kg crude protein and 54.1 g/kg crude lipid, which was identical to two *Momordica charantia* saponin-containing diets (Table 1). Dietary proteins were supplied by fish meal, chicken powder, soybean meal, and soybean protein concentrate. Soybean oil and fish oil were used as the main lipid sources, and cassava starch was used as the main carbohydrate source. The microcrystalline cellulose was equally diminished as the MS levels increased to ensure that the total volume is 100% for all formulations. All dry ingredients were sieved *via* an 80-mesh sieve. The ingredients were blended *via* a mixing machine. Subsequently, fish oil, soybean oil, and water were added to accomplish suitable viscosity. After the aforementioned processing, the mixed ingredients were extruded into pellets *via* a single-screwed extruder (EXT50A, Yang Gong Machine, China) and dried for 4 h in a ventilated oven at 55°C to approximately obtain 10% dietary moisture. Then, the dry pellets were smashed into proper pellet size (3.0 × 2.0 mm) for common carp to form the experimental sinking pellet and readily stored at −20°C until further use.

**TABLE 1:** Formulation and proximate chemical composition of the basal diets (g/kg dry matter).

Ingredient	HS	HS_MS1	HS_MS2
Fish meal^a^	35	35	35
Chicken powder^a^	45	45	45
Soybean meal^a^	400	400	400
Soybean protein concentrate^a^	120	120	120
Carboxymethylcellulose sodium	10	10	10
Microcrystalline cellulose	17	15.4	10.6
Cassava starch	300	300	300
Fish oil	20	20	20
Soybean oil	20	20	20
Choline chloride	5	5	5
Monocalcium phosphate	20	20	20
Vitamin premix^b^	3	3	3
Trace mineral premix^c^	2	2	2
Methionine	3	3	3
*Momordica charantia* saponins (MS)	0	1.6	6.4
Chemical composition			
Moisture	106.4	106	106
Crude protein	304.8	304.9	304.9
Crude lipid	54.1	54.2	54.2
Crude ash	72.8	73.0	72.8
Crude fiber	52.6	52.3	52.6
Nitrogen-free extract (NFE)^d^	409.3	409.6	409.6

^a^ Fish meal: crude protein 670.0 g/kg dry matter and crude lipid 48.5 g/kg dry matter; Chicken powder: crude protein 660.0 g/kg dry matter and crude lipid 130.0 g/kg dry matter; Soybean meal: crude protein 440.0 g/kg dry matter and crude lipid 15.0 g/kg dry matter; Soybean protein concentrate: crude protein 630.0 g/kg dry matter and crude lipid 5.0 g/kg dry matter. The aforementioned raw materials were purchased from Hehe Feed co. LTD., Harbin, China.

^b^ The vitamin premix supplied by Guangdong Hyint Biotechnology Group Co. Ltd. provided the following per kg of the diet: VA 8000 IU; VC 500 mg; VD3 3000 IU; VE 60 mg; VK3 5 mg; VB2, 30 mg; VB6 15 mg; VB12 0.5 mg; choline chloride 5000 mg; nicotinic acid 175 mg; D-biotin 2.5 mg; inositol 1000 mg; folic acid 5 mg; and pantothenic acid 50 mg.

^c^ The mineral premix supplied by Guangdong Hyint Biotechnology Group Co. Ltd. provided the following per kg of the diet: zinc (Zn) 25 mg; copper (Cu) 3 mg; iron (Fe) 25 mg; manganese (Mn) 15 mg; iodine (I) 0.6 mg; cobalt (Co) 0.1 mg; and selenium (Se) 0.4 mg.

^d^ Nitrogen-free extract (NFE) (%) = 100-[Moisture (%) − Crude protein (%) − Crude lipid (%) − Crude ash (%) − Crude fiber (%)].

Juvenile common carp were provided by the commercial farm (Chengdu, Sichuan). After acclimatized to laboratory conditions with a commercial feed (30% protein, Guangzhou Haid Group Co., LTD., China) for 14 days, 225 common carp of similar sizes (6.43 ± 0.02 g) were evenly and randomly allotted into nine indoor aquariums (capacity: 200 L), which were equipped with a recirculating system and an auto-supplemented oxygen system. Each diet was randomly distributed to triplicate aquarium and hand-fed to all the common carp at the ratio of 7%–8% of the body weight three times a day (8:00 a.m., 13:00 p.m., and 17:30 p.m.).

After 8-week experiment, common carp were adequately starved for 24 h. Six fish in each aquarium were deeply anesthetized with 100 mg kg^-1^ MS-222 (Zheng et al., 2021) and instantly dissected. The livers of common carp were obtained, promptly frozen in liquid nitrogen, and preserved at −80 °C for the follow-up experiment.

### RNA extraction

Total RNA was isolated from the liver samples using RNAiso Plus following the manufacturer’s recommendations. Total RNA integrity and purity were evaluated using a 2100 Bioanalyzer (Agilent Technologies, Santa Clara, CA, USA) and NanoDrop ND2000 spectrophotometer (Thermo Fisher Scientific, Wilmington, MA, USA). Only high-quality RNA samples (OD260/280 within 1.8–2.2, OD260/230 ≥ 2.0, and RIN≥7, 28S:18S ≥ 1.0, >3 μg) were used to construct the sequencing library.

### Small RNA sequencing and analysis

Library preparation for small RNA sequencing: After RNA extraction, a total amount of 3 μg total RNA per sample was used as the input material for the construction of a small RNA library. Sequencing libraries were generated using NEBNext^®^ Multiplex Small RNA Library Prep Set for Illumina^®^ (NEB, USA.) referring to manufacturer’s recommendations. NEB 3′-SR adaptor was directly and specifically ligated to 3′-end of miRNA. After the 3′-ligation reaction, the SR RT primer hybridized to the excess of 3′-SR adaptor and transformed the single-stranded DNA adaptor into a double-stranded DNA molecule. 5′-end adaptor was ligated to 5′-ends of miRNAs. Then, first strand cDNA was synthesized using M-MuLV reverse transcriptase (RNase H-). Polymerase chain reaction (PCR) amplification was conducted using LongAmp Taq 2X Master Mix, SR Primer for illumina, and index (X) primer. PCR products were purified on 8% polyacrylamide gel (100V, 80 min). DNA fragments corresponding to 140–160 bp (the length of small non-coding RNA plus the 3′- and 5′-adaptors) were recovered and dissolved in 8 μL elution buffer. Library quality was assessed on the Agilent Bioanalyzer 2100 system using DNA high-sensitivity chips. After cluster generation, the library preparations were sequenced on an Illumina platform and 50 bp single-end reads were generated.

Read mapping and transcriptome assembly: The raw paired end reads were trimmed and quality controlled by SeqPrep (https://github.com/jstjohn/SeqPrep) and Sickle (https://github.com/najoshi/sickle) with default parameters. Then, clean reads were separately aligned to the reference genome with the orientation mode using HIASAT (https://ccb.jhu.edu/software/hisat2/in dex. shtml) software. The mapped reads of each sample were assembled by StringTie (https://ccb.jhu.edu/software/stringtie/index.shtml?t=example) in a reference-based approach.

Identification of miRNAs: Low-quality bases (Sanger base quality of <20) of the 3’ end were trimmed using in-house perl scripts, and then the sequencing adaptors was removed using FASTX toolkit software (http://hannonlab.cshl.edu/fastx_toolkit/). All identical sequences of sizes ranging from 18 to 32 nt were counted and eliminated from the initial data set. The assembled unique sequences were used for a Basic Local Alignment Search Tool (BLAST) search of the Rfam database, version 10.1 (http://rfam.sanger.ac.uk/), to remove non-miRNA sequences (rRNA, tRNA, and snoRNA*.*). Bowtie (http://bowtie-bio.sourceforge.net/index.shtml) was used to annotate the chromosomal location against the reference genome data. Through a BLAST search of the miRbase, version 21.0 (http://www.mirbase.org/), the perfectly matched sequences were used to count and analyze the known miRNA expression profile. The characteristics of the hairpin structure of miRNA precursors can be used to predict novel miRNA. The available software MIREAP (Friedlander et al., 2012) or miRDeep2 was used to predict novel miRNA, the Dicer cleavage site, and the minimum free energy of the small RNA tags unannotated in the former steps. At the same time, in-house scripts were used to obtain the identified miRNA base bias on the first position with certain length and on each position of all identified miRNA. The expression level of each miRNA was calculated according to the transcripts per million reads (TPM) method. Significant differentially expressed (DE) miRNAs were extracted with a false discovery rate (FDR) < 0.05 and |log2FC| >1 by DESeq2.

### Prediction and analysis of the target genes of the miRNAs

RNAhybrid and miRNAda software programs were used for target gene prediction, and the overlapping genes between the two algorithms were regarded as the target genes. To understand the possible functions and classification of the predicted target genes, the predicted target genes were compared with six major databases: Gene Ontology (GO), Kyoto Encyclopedia of Genes and Genomes (KEGG), Clusters of Orthologous Groups (COG), the NCBI non-redundant protein (NR), and Swiss-Prot protein families (Pfam) to obtain comprehensive functional annotation information of target genes.

### mRNA sequencing and analysis

After RNA extraction, the concentration of RNA samples was adjusted to 1000 ng/ul. Inverse transcription was conducted using TaKaRa PrimeScriptTM RT Reagent Kit with gDNA Eraser (Perfect Real Time) (Code No: RR047A) (Dalian Takara Company). Hereupon, based on the Illumina NovaSeq 6000 sequencing platform, all mRNAs transcribed from the liver at a certain period were sequenced by Shanghai Majorbio Bio-pharm Technology Co., Ltd. The Illumina Truseq™ RNA Sample Prep Kit method was used for library construction. After obtaining the read counts of genes/transcripts by gene expression analysis, DESeq2, DEGseq, or edgeR can be used to analyze the differential expression of genes between samples or groups of multiple (≥2) items to identify the differentially expressed genes. The expression levels of the genes in each library were analyzed with the FPKM method. Genes with fold change (FC) ≥ 2 (|log 2 FC| ≥ 1) and FDR<0.05 were assigned as differentially expressed. GOATOOLS software was used for GO enrichment analysis of genes, and R script was used to perform KEGG PATHWAY enrichment analysis on genes/transcripts. The GO function or KEGG PATHWAY was considered to be significantly enriched when the adjusted *p*-value (P adjust) was <0.05 using Fisher’s exact test.

### Data processing and analysis

Statistical analyses of data involved in significance analysis were performed using SPSS 23.0 (SPSS Inc., Chicago, IL, U.S.A.). Comparisons between groups were performed by one-way analysis of variance with Duncan’s *post hoc* test. Results were considered statistically significant when *p* < 0.05.

## Results and analysis

### Analysis of miRNA sequencing of the common carp liver

Through sequencing analysis, 11,451,779, 12,715,467, and 10,824,276 original data were, respectively, obtained in HC, HS_MS1, and HS_MS2 groups. After removing sequences whose length was not between 18 nt and 32 nt, 10260715, 10973698, and 9657671 pure and usable sequences were gained for subsequent analysis in the three groups, respectively (Table 2).

**TABLE 2:** Sequencing data of microRNA libraries (n = 6).

	Raw read	Clean read		Useful read (18 nt–32 nt)	
	Quantity	Quantity	Proportion/%	Quantity	Proportion/%
HC	11451779	10999043	96.04	10260715	89.59772128
HS_MS1	12715467	12059887	94.82	10973698	86.39104175
HS_MS2	10824276	10352985	95.64	9657671	89.22753886

**FIGURE 1:**
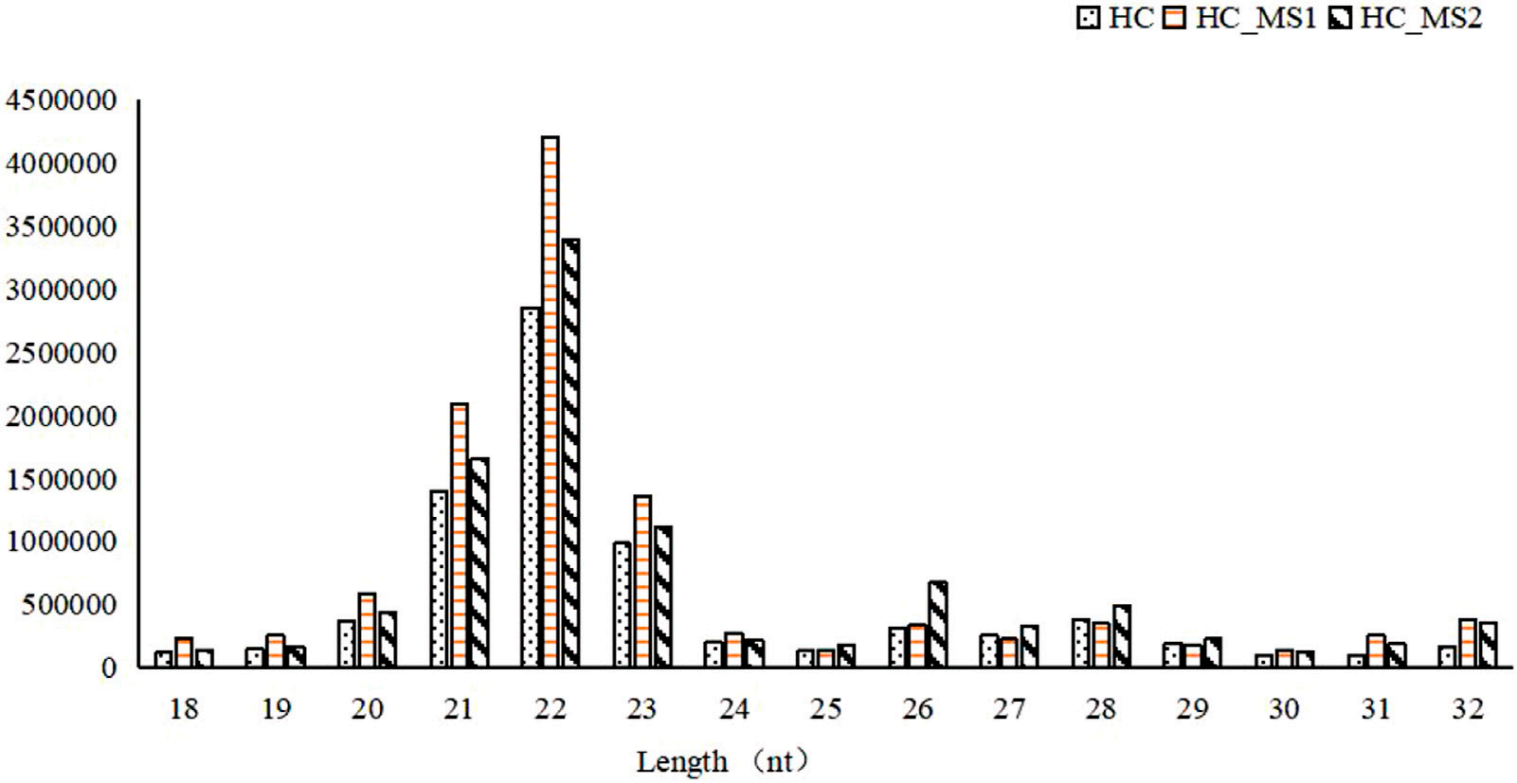
Length distribution of small RNAs in the three groups (n = 6).

Most of the library lengths of all miRNAs in three groups were distributed between 21 and 23 nt, which was consistent with the length of the product after Dicer digestion, and were the typical length of known miRNA. Among them, 22-nt sequences were the most common (24.87% of the library small RNA of the HC group, 33.04% of the library small RNA of the HS_MS1 group, 31.34% of the library small RNA of the HS_MS2 group). Although the length distribution of small RNA was similar among the three groups, the proportion of small RNA was different to some extent. Therefore, it was speculated that MS in high-carbohydrate diet could potentially regulate the carbohydrate metabolism of the common carp liver (Figure 1).

As shown in Table 3, the pure sequences that could be located to the whole genome of common carp were compared with GenBank and Rfam databases. Studies showed that the proportion of rRNA as a quality control standard should be less than 40%. In our experiment, the proportion of rRNA in the three groups was 3.60%, 5.34%, and 3.84%, respectively, indicating that the sample quality is eligible. Meanwhile, other non-coding RNAs except miRNAs accounted for a small proportion in the library of the three groups. After these non-coding RNAs were filtered out, there were 6148380, 6428180, and 5215385 sequences in the three groups for subsequent miRNA analysis, respectively.

**TABLE 3:** Small RNA annotations and quantity distribution in the three groups (n = 6).

Category	HC		HS_MS1		HS_MS2	
	Quantity	Proportion/%	Quantity	Proportion/%	Quantity	Proportion/%
Known miRNA	4510721	63.67	4532901	60.21	3771572	62.46
Novel miRNA	1273921	17.88	1774761	23.34	1354460	22.42
rRNA	257,625	3.60	406,365	5.34	232,325	3.84
tRNA	36,807	0.53	41,017	0.57	67,811	1.13
snoRNA	445	0.01	533	0.01	438	0.01
snRNA	111	0.00	106	0.00	343	0.00
Repeat	29,555	0.42	28,040	0.37	18,546	0.31
Exon	392,561	5.58	428,388	5.75	337,733	5.59
Intron	196,435	2.79	212,116	2.82	166,626	2.76
Unknown	363,738	5.50	120,518	1.58	89,353	1.48
For analysis	6148380	87.06	6428180	85.13	5215385	86.36

**FIGURE 2:**
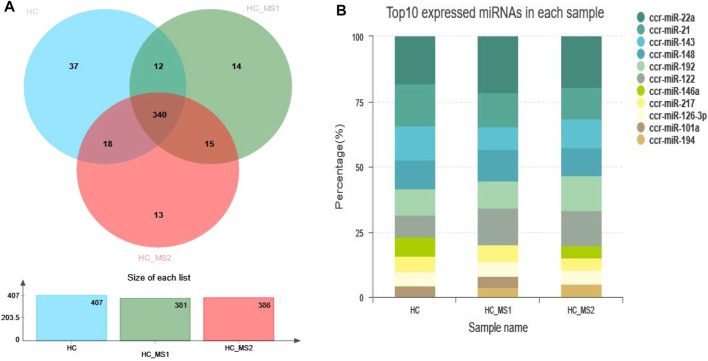
Sequence quantity of known miRNAs and the 10 known miRNAs with the most abundant expression in three libraries (n = 6).

After small RNA annotation and screening, the remaining sequences were compared with the known miRNA sequences of common carp listed in miRBase 21.0. A total of 407, 381, and 386 known miRNAs were identified in the three libraries, respectively. Among them, 37 miRNAs were only found in the library of the HC group, 14 miRNAs were only found in the suitable group library of the HS_MS1 group, and 13 miRNAs were only found in the library of the HS_MS2 group (Figure 2A). The 10 known miRNAs with the most abundant expression in the libraries of three groups are presented in Figure 2B. It is worth mentioning that the 10 known miRNAs with the most abundant expression in the three libraries were basically the same.

**FIGURE 3:**
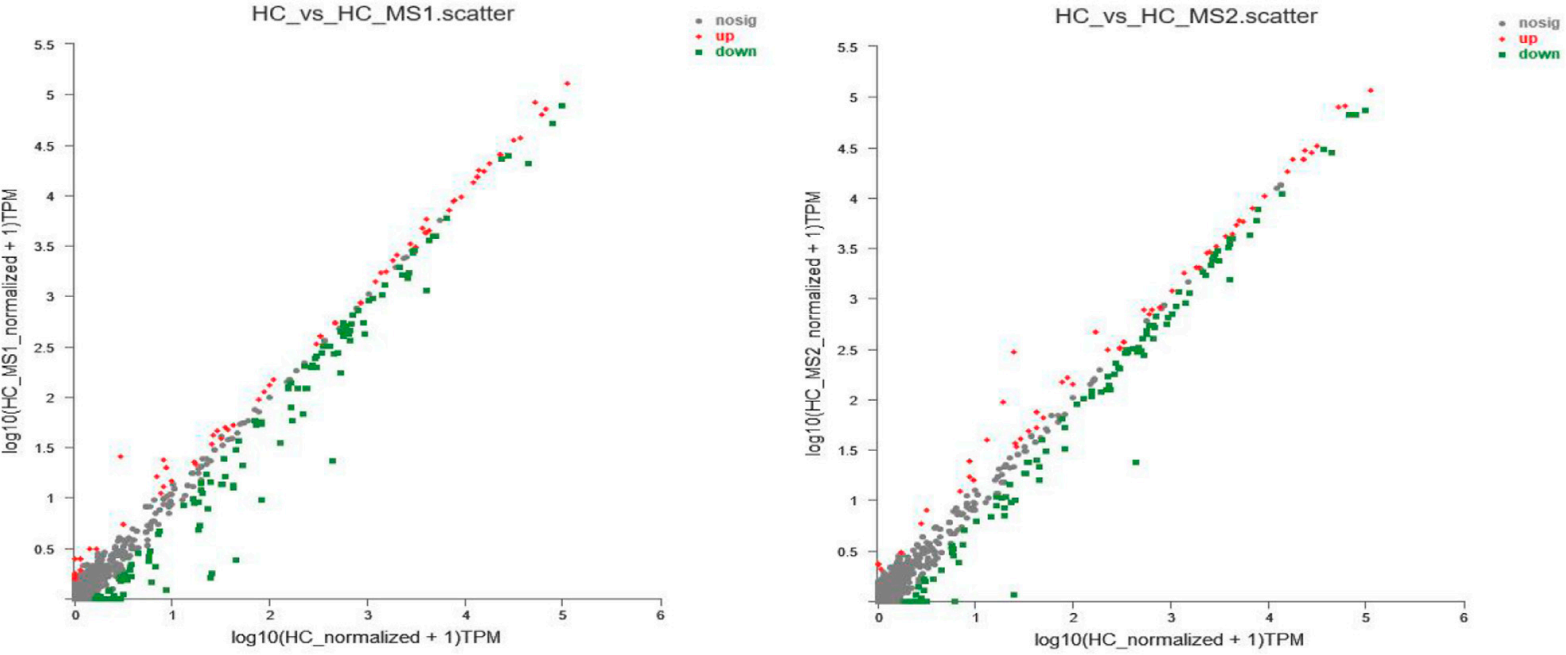
Scatter plot of miRNA expression levels of the three libraries. The abscissa represents log_10_ (miRNA expression level of the standardized NFD group + 1), the ordinate represents log_10_ (miRNA expression level of standardized the HFD group + 1), and each point represents one miRNA. Red represents significant upward revision, and green represents significant downward revision (n = 6).

In order to further study the influence of MS on miRNA expression spectrum, analysis of miRNA expression difference was conducted. As shown in Figure 3A, compared with the HC group, the expression of 85 miRNAs (31 known miRNAs and 54 unknown miRNAs) were upregulated, and 157 miRNAs (65 known miRNAs and 92 unknown miRNAs) were downregulated in the HS_MS1 group. In the HS_MS2 group, the expression of 67 miRNAs (33 known miRNAs and 34 unknown miRNAs) were upregulated, and 157 miRNAs (63 known miRNAs, 84 unknown miRNAs) were downregulated.

### Target gene prediction for significantly differentially expressed known miRNAs

**FIGURE 4:**
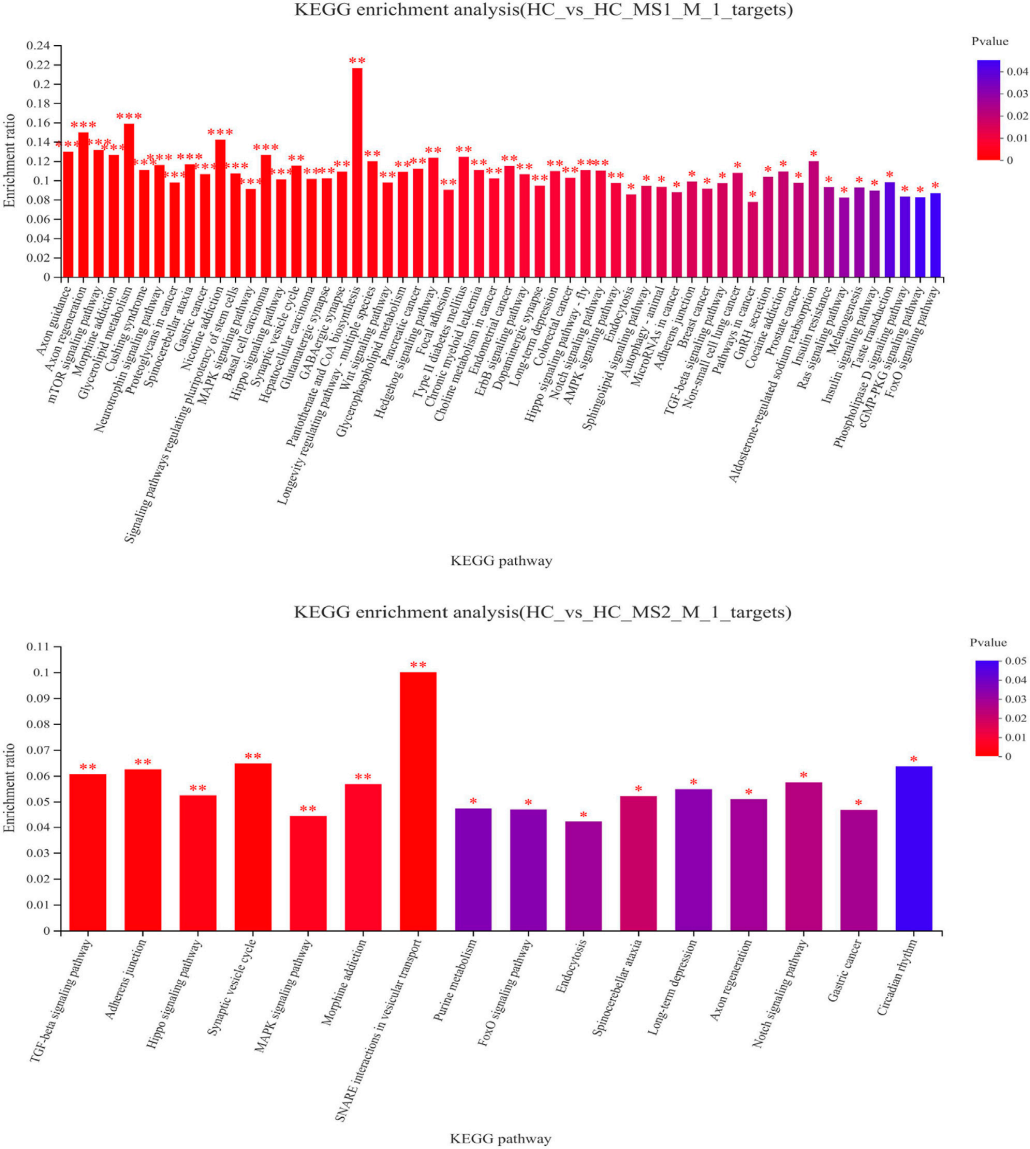
KEGG enrichment analysis of differentially expressed candidate target genes of known miRNAs in the liver (n = 6).

To understand the physiological processes and pathways that these identified miRNAs might regulate, the potential target genes were predicted through KEGG pathway analysis. KEGG pathway analysis revealed that the main metabolic pathways in which candidate target genes were involved were lipid metabolism and carbohydrate metabolism (Figure 4A). Compared to the HC group, 58 pathways in the HS_MS1 group were significantly enriched (*p* < 0.05), including insulin signaling pathway, insulin resistance, mTOR signaling pathway, type II diabetes mellitus, and MAPK signaling pathway, which are closely correlated with carbohydrate metabolism and insulin resistance of the liver. However, only 16 pathways in the HS_MS2 group were significantly enriched (*p* < 0.05), and only the MAPK signaling pathway was associated with insulin resistance (Figure 4).

Through seeking for the reported genes related to insulin signaling pathway and insulin resistance in the target miRNAs and combining the results of KEGG enrichment analysis, 10 significantly differentially expressed miRNAs in HC and HS_MS1 groups, including one upregulated and nine downregulated miRNAs, were identified, whereas 11 significantly differentially expressed miRNAs in HC and HS_MS2 groups, including four upregulated and six downregulated miRNAs, were identified (Table 4 and Table 5). These miRNAs may not only be involved in the regulation of insulin signaling pathway and insulin resistance in common carp but also be the markers for the liver in MS therapy for the remission of insulin resistance.

**TABLE 4:** Differential expression of miRNAs in HC and HS_MS1 groups associated with insulin signaling pathway and insulin resistance of common carp (n = 6).

miRNA name	Log2^FC^(HS_MS1/HS)	*p*-value	Significant	Regulate	HC	HS_MS1
ccr-miR-10b	−0.239290418	1.93E-25	Yes	Down	522.0925333	438.2769
ccr-miR-122	0.657974485	0	Yes	Up	52,372.03363	83,491.76903
ccr-miR-143	−0.654959485	0	Yes	Down	79,410.908	51,305.91907
ccr-miR-146a	−1.118448091	0	Yes	Down	44,268.8926	20,617.48717
ccr-miR-155	−1.642536895	0	Yes	Down	530.3959	171.2446667
ccr-miR-16c	−0.097911691	4.75E-19	Yes	Down	2132.527933	1946.772633
ccr-miR-200a	−0.346154361	0	Yes	Down	4931.0393	3950.1305
ccr-miR-29a	−0.409782408	3.06E-78	Yes	Down	595.8598667	447.2451333
ccr-miR-34	−0.119543827	0.000492397	Yes	Down	221.7779667	198.2857667
ccr-miR-375	−1.897256718	0	Yes	Down	4005.150467	1118.8686

**TABLE 5:** Differential expression of miRNAs in HC and HS_MS2 groups associated with insulin signaling pathway and insulin resistance of common carp (n = 6).

miRNA name	Log2^FC^(HS_MS1/HS)	*p*-value	Significant	Regulate	HC	HS_MS1
ccr-miR-10b	0.620911371	9.85E-197	Yes	Up	522.0925333	771.6064
ccr-miR-122	0.593702309	0	Yes	Up	52,372.03363	79,858.56307
ccr-miR-143	−0.251891619	0	Yes	Down	79,410.908	66,825.88577
ccr-miR-146a	−0.635194462	0	Yes	Down	44,268.8926	27,888.13847
ccr-miR-155	−0.957112528	2.69E-278	Yes	Down	530.3959	268.1136
ccr-miR-16c	−0.193557903	2.44E-61	Yes	Down	2132.527933	1842.340867
ccr-miR-200a	0.305772174	0	Yes	Up	4931.0393	5970.175367
ccr-miR-29a	−0.135747397	5.67E-10	Yes	Down	595.8598667	539.2821
ccr-miR-34	0.517876072	4.26E-58	Yes	Up	221.7779667	310.2684333
ccr-miR-375	−1.406625562	0	Yes	Down	4005.150467	1527.210033

The 252 target genes corresponding to 10 significantly differentially expressed miRNAs were predicted by miRDB online databases and are presented in Table 6. Based on biopathway enrichment analysis of the 252 target genes using the KEGG classical biopathway database, it was revealed that gsk3bb, pik3r1, and pik3r3b were enriched in PI3K-AKT signaling pathway, insulin signaling pathway, and insulin resistance.

**TABLE 6:** Target genes of miRNAs associated with insulin signaling pathway and insulin resistance of common carp.

Serial number	miRNA	Target gene
1	ccr-miR-10b	adam15, arhgap17b, chl1b, dpysl5a, gpr26, kcna4, pabpc4, and trioa
2	ccr-miR-122	actr2a, b4galt5, ccdc17, csmd3b, dcp1a, dlb, eloca, fam117ba, fam13b, gria4b, hipk3b, lnx1, ppp1r9ala, ptprz1a, rab3il1, rbm47, rsph4a, shtn1, slc8a4b, tgfa, twist2, ube2g2, usp43a, wnt3, yes1, and znf296
3	ccr-miR-143	adgrl1a, arnt, ca21h5orf15, calm3a, cdc42ep4b, chmp2ba, dok1b, emx1, fbxl22, gje1, hmcn2, lrrc56, nrg3b, pals2a, **pik3r3b**, rsf1b.1, sec16b, sharpin, slc16a2, smtnb, stxbp2, tfap4, tmed4, ttll1, and txnl4b
4	ccr-miR-146a	actn4, ago2, alkal2b, bmp2b, cfap45, dpp6a, enpp7.2, fancm, gla, hoxc4a, htr4, kif5bb, klhl11, lbr, magixa, mtfp1, nifk, oxnad1, qki2, rab32a, rnf180b, rnf220a, rybpa, slc24a6a, tfdp1a, trps1, and zfand6
5	ccr-miR-155	acvr1bb, apobec2a, atad2b, chata, cux1a, faxdc2, g2e3, hapln1b, pabpc4, pla2r1, rab33a, sall1b, and sulf2b
6	ccr-miR-16c	adam11, alx4a, ankfy1, asb1, cacna1fb, camk2d1**,** cdc42ep2, cdk5rap3, chka, ctsll, dnajb12b, eef2k, fam199x, fgd4b, galnt13, gria4b, **gsk3bb**, igfbp2b, kalrna, kcnc3b, kcnh3, klhl38a, lekr1, lrrc4ba, mfsd2ab, mrps5, mtf2, slc7a3b, ssh1b, stk35l, svep1, sys1, tnmd, trim13, ubr3, vti1a, zfyve28, and zhx3a
7	ccr-miR-200a	ca8h1orf174, cab39, cd276, dhx34, dipk1ab, dpysl2a, fam222bb, fgf1b, galnt13, hbegfa, hic2, hoxb9a, igfbp2b, il10, jag1a, kcna4, khdrbs2, lars2, mamdc2a, mmrn2b, negr1, nudt17, osbpl3b, pdk4, phf14, prickle2a, prlra, rargb, rhbdd2, rnpepl1, slc4a10a, tecpr1a, tfr1b, ywhag2, zgpat, and znf800a
8	ccr-miR-29a	ago3b, akap1b, arhgap39, b4galt5, cbx5, cdc14aa, cdh15, chl1b, col7a1, cpne3, crfb12, cyth3b, dnajb6b, ehmt1b, fam91a1, fbln2, ing4, irf2, kif9, ndor1, nkx3-2, **pik3r1,** pparaa, ptprq, ripor3, shoc2, syde2, tgfbr1b, tp63tp73, ubxn6, wfdc1, zfand2a, znf827, and zzef1
9	ccr-miR-34	abca1a, ackr4b, agtr1b, akt3a, b3gnt5a, cadps2, cdc14aa, cnih3, cntn1a, crfb1, ctdsp1, dock4, emx1, erf, frmd8, glb1l2, gucy2d, hoxa11b, jazf1a, klf7b, lrmda, lrrtm4l2, mmp9, nfasca, notch3, npas4l, rab43, rasa1a, slc13a3, sptbn4a, tgfbrap1, tmem102, vti1a, xab2, and znf648
10	ccr-miR-375	abraxas2, c2cd2l, camsap3, cdkn1bb, faf1, irf3, slc6a11a, and st8sia5

### Transcriptome sequencing of the common carp liver

About 43.19–49.36 M total raw reads were produced for three libraries. All the obtained RNA-seq data have been submitted to the NCBI Sequence Read Archive (GCF_018340385.1; Reference genome source: https://www.ncbi.nlm.nih.gov/genome/?term = Cyprinus_carpio). RNA-seq yielded 43,194,863–49,364,038 clean reads with an average Q20 bases at approximately 98.40%, after low-quality reads, adaptors, and poly-Ns were filtered out. Among the three groups, approximately 91.75%–93.08% clean reads were successfully mapped in the *Cyprinus carpio* reference genome source (Table 7).

**TABLE 7:** Sequencing data of mRNA libraries (n = 6).

	Raw read	Clean read		Q20 (%)	GC content (%)
		Quantity	Mapping proportion/%		
HC	47151389	46725101	92.93	98.41	48.93
HS_MS1	49364038	49001715	91.75	98.48	49.62
HS_MS2	43194863	42819838	93.08	98.40	49.00

**FIGURE 5:**
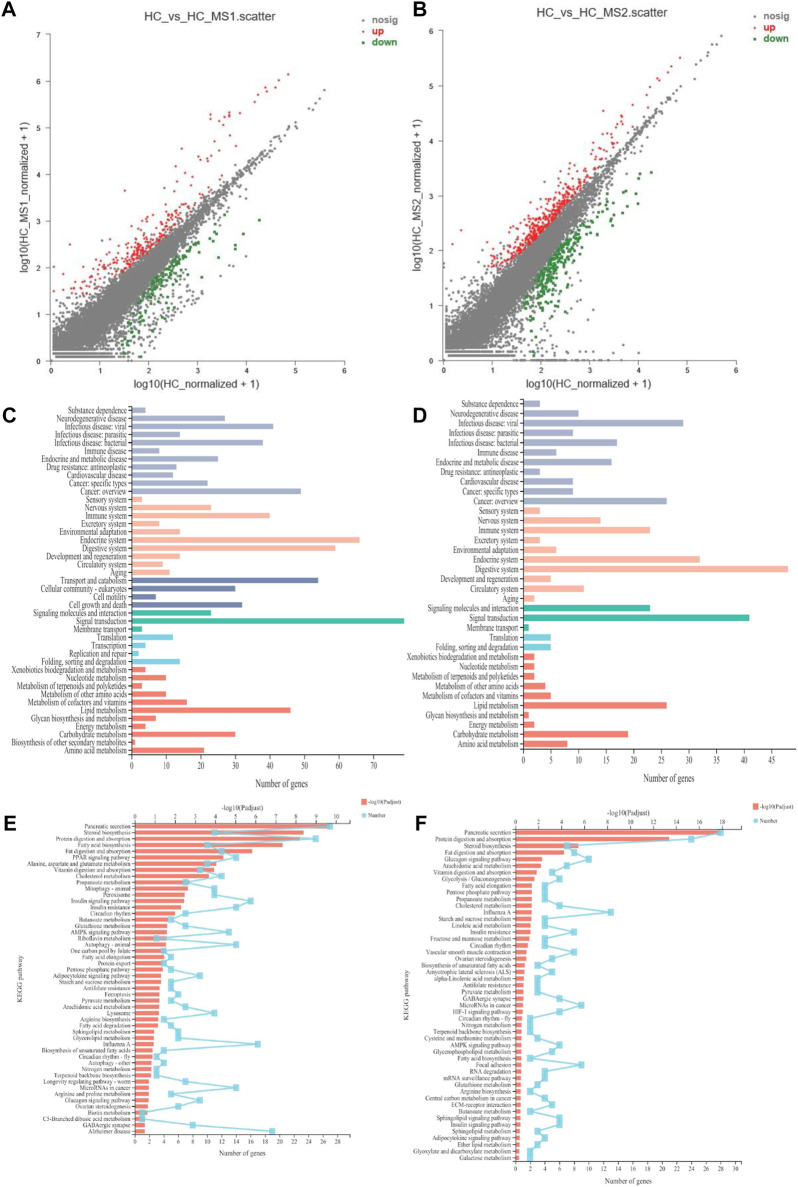
Scatter diagram of differentially expressed genes in RNA-seq **(A,B)**. KEGG pathway category of differentially expressed genes **(C,D)**. KEGG pathway enrichment in RNA-seq **(E,F)** (n = 6).

Figure 5A shows the DEG situation between the HC and HS_MS1/HS_MS2 groups. A total of 558 genes were determined to be significantly differentially expressed between the HC and HS_MS1 groups. Among those genes, 238 were upregulated and 693 were downregulated in the HS_MS1 group. Additionally, a total of 1,634 genes were determined to be significantly differentially expressed between the HC and HS_MS1 groups. Among those genes, 401 were upregulated, and 220 were downregulated in the HS_MS1 group.

In terms of KEGG annotation analysis, DEGs between the HC and HS_MS1/HS_MS2 groups were distributed to 44 KEGG terms, of which signal transduction, endocrine system, digestive system, transport and catabolism, lipid metabolism, immune system, and carbohydrate metabolism were mainly enriched. Further KEGG enrichment analysis revealed that 50 pathways with Q-value ≤ 0.05 were significantly enriched. The comparison between the HC and HS_MS1 groups revealed that markedly enrichments of insulin signaling pathway, insulin resistance, and AMPK signaling pathway were closely related to the carbohydrate metabolism, whereas the comparison between the HC and HS_MS2 groups revealed that markedly enrichments of glycolysis/gluconeogenesis and insulin resistance were closely related to the carbohydrate metabolism. These results indicated that these genes may be bound up with the glucose homeostasis in common carp.

### Expression verification of the selected genes related to insulin signaling pathway and insulin resistance of common carp based on RNA-seq

**FIGURE 6:**
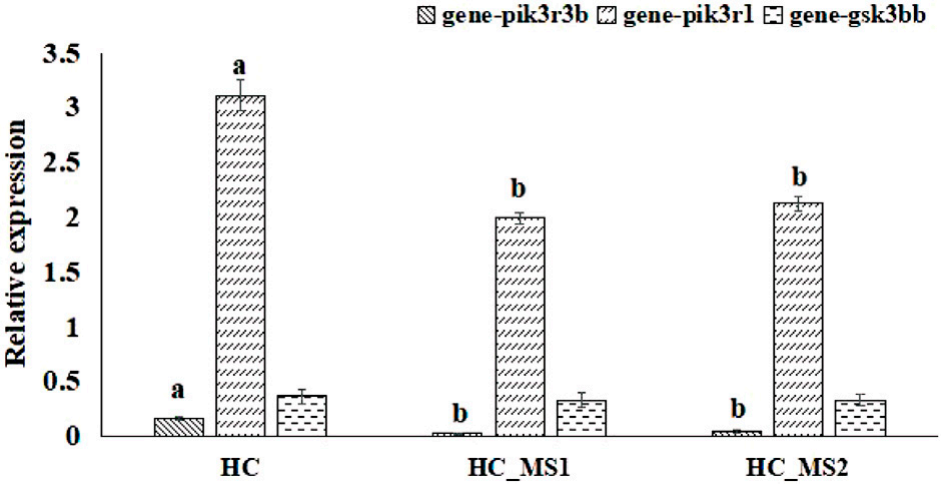
Quantitative expression verification of the selected genes related to insulin signaling pathway and insulin resistance of common carp based on RNA-seq (n = 6).

According to the biopathway enrichment analysis of the 252 target genes using the KEGG classical biopathway database, the relative expression levels of gsk3bb, pik3r1, and pik3r3b were analyzed using RNA-seq. As shown in Figure 6, compared to the HC group, a significant decrease in the relative expression levels of pik3r1 and pik3r3b were observed in HS_MS1 and HS_MS2 groups (*p* < 0.05), whereas no marked differences were observed in gsk3bb expression (*p* > 0.05).

## Discussion

The regulatory mechanism of fish response to high-glucose intake or high-carbohydrate diets is multi-phase, complicated, and puzzled. Conversely, however, our understanding of this mechanism has been still mainly limited to physiological and biochemical changes *in vivo* (Hemre et al., 2002; Kamalam et al., 2017). Nowadays, the accelerated development in next-generation sequencing (NGS) provides a new train of thought and method for a deeper comprehension of the specific mechanism of fish response to high-glucose intake or high-carbohydrate diets at a molecular level. Analogously, the mechanism of functional nutrient material regulating fish carbohydrate metabolism has been still maintained at physiological and biochemical levels (Xu et al., 2017; Xu et al., 2021). Herein, this study emphasized to describe the typical differences in miRNA-seq and RNA-seq for common carp, which were, respectively, fed with high-starch diets and high-starch diets containing *Momordica charantia* saponins. These data offer a new perspective of regulating and improving the carbohydrate metabolism of common carp.

Existing studies preliminarily illustrated that miRs may play an important role in carbohydrate metabolism in fish. Studies on *Megalobrama amblycephala* revealed that miR-34a could not only participate in the regulation of glucose metabolism by suppressing the related gene expression of the AMPK signaling pathway (Miao et al., 2018) but also affect the digestive absorption of fish fed with a high-carbohydrate diet through sirtuin 1 (Sirt1)/forkhead box O1 (FoxO1 axis) (Miao et al., 2019). Rasal et al. (2020) pointed out that differentially expressed miR-22, miR-122, miR-365, miR-200, and miR-146 closely correlated with carbohydrate metabolism were obtained when *Labeo rohita* (Hamilton, 1822) were fed with a high-carbohydrate diet for 45 days. In view to carbohydrate metabolism of common carp, the only conducted study indicated that common carp could downregulate the expression of miR-122, miR-30b, and miR-15b-5p to improve the liver carbohydrate metabolism ability in response to cold and heat stress (Sun et al., 2019). Our study revealed that under the intervention of *Momordica charantia* saponin administration, the 10 identified differentially expressed miRNAs in the common carp liver, namely, ccr-miR-10b, ccr-miR-122, ccr-miR-143, ccr-miR-146a, ccr-miR-155, ccr-miR-16c, ccr-miR-200a, ccr-miR-29a, ccr-miR-34, and ccr-miR-375, have been proven to play a role in regulating the insulin signal transduction pathway and carbohydrate metabolism (Chakraborty et al., 2014; Wei et al., 2020; Chen et al., 2021) and have the potential to be underlying biomarker candidates for alleviating insulin resistance in the liver of common carp fed with high-carbohydrate diet.

After the target genes were predicted by the KEGG classical biopathway database, we found that from the perspective of the correlation between enriched pathway and insulin resistance, some differentially expressed candidate target genes between HC and HS_MS1 groups were markedly enriched in the insulin signaling pathway, insulin resistance, mTOR signaling pathway, type II diabetes mellitus, and MAPK signaling pathway, whilst these candidate target genes between HC and HS_MS2 groups were markedly enriched in the MAPK signaling pathway. Interestingly, based on RNA-seq results, the identified differentially expressed mRNAs in the common carp liver derived from the comparison between HC and HS_MS1 groups were mainly concentrated in the insulin signaling pathway and AMPK signaling pathway. These comprehensive and consistent results may suggest that the existence of appropriate *Momordica charantia* saponins in high-starch diets for common carp would adjust the insulin signaling pathway to remit insulin resistance through activating or restraining certain miRs (Zhao et al., 2020). For fish, glucose homeostasis is the key to maintaining the health status. The liver maintains the glucose homeostasis depending on regulating gluconeogenesis and glycogen synthesis through the insulin signaling pathway. The insulin signaling pathway in the liver is triggered by the binding of insulin and insulin receptor giving a signaling to insulin receptor substrates, which could activate a variety of downstream pathways to regulate liver function (Luo and Zhuo, 2018). Therein, the PI3K/AKT/GLUTS/GSK-3β pathway is a major pathway of insulin signaling transduction and an important pathway of blood glucose regulation, which is closely associated with insulin resistance-related diseases (Xiao and Ren, 2020; Li et al., 2017). Furthermore, these target genes predicted by miRDB online databases based on KEGG classical biopathway can provide more explicit directions for insulin resistance in the common carp liver, especially the three genes, gsk3bb, pik3r1, and pik3r3b, which were highly correlated to the insulin signaling pathway.

Amongst the insulin signaling transduction pathways, key regulatory protein PI3K consists of regulatory subunits (p85α, p55α, and p50α encoded by pik3r1, p85β encoded by pik3r2, and p55γ encoded by pik3r3) and catalytic subunits (p110α and p110β encoded by pik3ca). Herein, the p85α subunit encoded by pik3r1 is the regulator of the PI3K signaling pathway and is responsible for inhibiting the activity of the p110 subunit. Therefore, the mutation and abnormal expression of pik3r1 gene will lead to the abnormality of the PI3K/AKT signaling pathway (Comb et al., 2012). pik3r1 can form a heterodimer with pik3ca and interact with insulin receptor substrate 1 (IRS-1) to activate the insulin signal transduction pathway. However, the excessive pik3r1 monomer can compete with the pik3R1/pik3ca heterodimer for the IRS-1-binding site, thus inhibiting the hypoglycemic activity of insulin (Chagpar et al., 2010). Franck et al. (2002) pointed out that knockdown of pik3r1 in diabetic mice could enhance the insulin sensitivity and glucose homeostasis, thereby ameliorating diabetes. In gibel carp, with the heterozygous deletion of pik3r1, the activity of the PI3K/AKT signaling pathway was markedly elevated (Huang et al., 2021). In addition, the pik3r3 mutant could also be able to downregulate the biological functions of insulin, such as inhibiting cellular glucose intake (Ueki et al., 2003). In the current study, there was no significant expression change in gsb3bb expression as measured by RNA-seq, whereas the expression of pik3r1 and pik3r3b was significantly and abnormally higher in the HC group than HS_MS1 and HS_MS2 groups, indicating that the insulin resistance of the liver in common carp fed with high-carbohydrate diets might result from the IRS anomaly or disturbance of PI3K activation induced by aberrant expression of pik3r1 and pik3r3, and *Momordica charantia* saponin administration in high-carbohydrate diets may be expected to adjust the two target genes to remit the liver insulin resistance of common carp.

Ascending to the upstream miRs, we found that pik3r1 is a potential target of miR-29a (positive regulation), and pik3r3b is a potential target of miR-143 (positive regulation). For terrestrial animals, miR-29a interferes with insulin signal transduction mainly through the PI3K/AKT pathway and plays a crucial role in the process of insulin resistance (Alimoradi et al., 2021; Paul et al., 2021). For Zucker diabetic rats, significant increment in miR-29a expression weakened the activation degree of the insulin signaling pathway, indicating that the miR-29a expression level was closely related to insulin resistance (Kurtz et al., 2014). Li et al. (2021) found that intravenous injection of miR-29a attenuated the liver insulin sensitivity of mice, and overexpression of miR-29a in β-cells of transgenic mice promoted the secretion of miR-29a and inhibited the activation of AKT. A study on db/db mice revealed that overexpression and knockout of miR-143 could inhibit the expression of the target gene oxysterol-binding protein-associated protein 8 (ORP8), resulting in insulin resistance (Jordan et al., 2011). In this study, ccr-miR-29a and ccr-miR-143 were markedly downregulated in HS_MS1 and HS_MS2 groups compared to the HC group. This result is in line with the finding of previous reports observing that whole grain highland barley could alleviate insulin resistance through enhancing the activity of the insulin signaling pathway induced by the decline in miR-29a expression (Deng et al., 2020). Henceforth, ccr-miR-29a and ccr-miR-143 might serve as a potential therapeutic biomarker in remitting the liver insulin resistance of common carp for functional substances such as *Momordica charantia* saponins.

The main purpose of our research is to explore and predict possible miRNA and mRNA biomarkers to improve the liver insulin resistance of common carp based on *Momordica charantia* saponin administration. In general, miRNAs play a negative regulatory role in gene silencing by complementary pairing with the 3′-non-coding region (3′-UTR) of target mRNA to degrade mRNA and inhibit protein translation (Bartel 2009). Additionally, miRNAs could also act on target genes through positive regulation and de-inhibition under specific conditions (Vasudevan 2012; Ma et al., 2016). In this study, the expression of pik3r1 and pik3r3 was downregulated in the HS_MS1 and HS_MS2 groups compared with that in the HC group, while the expression of miR-29a and miR-143 was also downregulated, signifying that the expression of miR-29a and pik3r1 and miR-143 and pik3r3 was likely to be positively targeting correlated. Instead, the underlying mechanism on *Momordica charantia* saponins ameliorating the liver insulin resistance of common carp might be to conduct the inhibition of ccr-miR-29a targeting pik3r1 or ccr-miR-143 targeting pik3r3. However, what needs to be emphasized is that there exist some limitations to our research. Of these, verification experiments at the animal individual level and cellular level need to be implemented as soon as possible. Hereafter, we will put emphasis on exploring the effects of miRs targeting mRNA on the insulin resistance *in vivo* or in hepatocyte of the common carp liver and pursuing more scientific evidence for *Momordica charantia* saponins relieving the insulin resistance of common carp in the future.

## Conclusion

This study further enhanced our understanding of miRNAs and mRNA in common carp (*Cyprinus carpio*). Through sequencing and data analysis, we identified 10 potential known miRNAs as candidates participating in modulating the liver insulin resistance and raised a presumption of the presence of ccr-miR-29a targeting pik3r1 or ccr-miR-143 targeting pik3r3 with likely crucial roles of *Momordica charantia* saponins in remitting the liver insulin resistance, so as to lay the foundation for improving the “congenital diabetic constitution” in common carp.

## Data Availability

The datasets presented in this study can be found in online repositories. The names of the repository/repositories and accession number(s) can be found at: https://www.ncbi.nlm.nih.gov/genome/?term=Cyprinus_carpio.
